# The Impact of Mortality Salience on Intergenerational Altruism and the Perceived Importance of Sustainable Development Goals

**DOI:** 10.3389/fpsyg.2018.01399

**Published:** 2018-08-03

**Authors:** Saiquan Hu, Xiaoying Zheng, Nan Zhang, Junming Zhu

**Affiliations:** ^1^Department of Psychology, School of Social Sciences, Tsinghua University, Beijing, China; ^2^Department of Marketing, School of Business, Nankai University, Tianjin, China; ^3^School of Public Policy and Management, Tsinghua University, Beijing, China

**Keywords:** sustainable development goals, mortality salience, intergenerational altruism, perceived importance, terror management theory

## Abstract

The Sustainable Development Goals (SDGs), consisting of 17 specific goals such as ending poverty, reducing inequality, and combating climate change, were proposed by the UN member states in 2014 for the ongoing UN agenda until 2030. These goals articulate the growing need for the international community to build a sustainable future. To progress and build a truly sustainable future requires not only the immediate support of individuals for the current SDGs, but also their personal long-term commitment to the needs of future generations (i.e., intergenerational altruism). Reminders of death can influence attitudes, motivation, and behavior in various aspects of our lives. In the current research, we thus explored whether reminding individuals of their own death will influence their intergenerational altruism and perceived importance of the SDGs. Using a three-condition (mortality salience vs. dentist visit vs. neutral) randomized experiment, we found that mortality salience led participants to place a higher priority on the needs of future generations only when compared to the neutral condition. Further, we conducted a factor analysis that generated two SDGs factors (socially related SDGs and ecologically related SDGs). We found that mortality salience reduced participants’ perceived importance of the socially related SDGs when compared to both the dentist visit and the neutral conditions, and mortality salience decreased participants’ perceived importance of the ecologically related SDGs only when compared to the neutral condition.

## Introduction

Sustainable development was first proposed by the Brundtland (1987, p. 43) report in as “development that meets the needs of the present without compromising the ability of future generations to meet their own needs.” It aims to balance the global linkages between issues of environmental degradation, socioeconomic problems associated with inequality and poverty, and the needs of future generations (Giddings et al., 2002). However, the concept of sustainable development cannot be used directly as a tool for addressing these global issues as it has a certain vagueness (Mebratu, 1998). To solve global problems that are halting sustainable development, it is necessary to operationalize this concept and transform it into related specific goals and precise action plans (Hopwood et al., 2005).

In 2000, the UN proposed the first set of operationalized goals—the Millennium Development Goals (MDGs), which was designed to address global issues such as ending poverty, ensuring food accessibility, and improving health (Sachs, 2012). Of these goals, socioeconomic problems rather than environmental issues were the main focus. In 2014 (1 year before the MDGs expired), the UN put forward the second set of operationalized goals—the Sustainable Development Goals (SDGs), which was designed to replace the MDGs and designate the UN agenda until 2030. As a new global agenda for promoting sustainable development, the SDGs not only inherited the commitments of the MDGs that focused on addressing socioeconomic issues, but also concentrated on issues related to governing the earth system and reducing the unsustainable practices of individuals, groups, and countries (Griggs et al., 2014).

More specifically, the SDGs consist of 17 goals: ending poverty (Goal1) and hunger (Goal2); promoting well-being (Goal3) and quality education (Goal4); achieving gender equality (Goal5); ensuring sustainable water (Goal6) and affordable energy (Goal7); promoting decent work (Goal8) and inclusive industrialization (Goal9); reducing inequalities between countries (Goal10); building sustainable cities (Goal11) and encouraging sustainable consumption (Goal12); combating climate change (Goal13); conserving marine life (Goal14); promoting sustainable ecosystems (Goal15) and inclusive societies (Goal16); and revitalizing global partnerships (Goal17). These goals articulate definite needs for the international community to build a sustainable future and were adopted by all countries in 2015 (United Nations, 2015).

Current literature on the SDGs has primarily focused on the content analysis of these goals (Davies, 2015; Gupta and Vegelin, 2016), the principles that should be followed when implementing these goals (Hajer et al., 2015), and the integrated dynamic method to evaluate the implementation effectiveness of these goals (Costanza et al., 2016). Although these research efforts are important for making SDGs implementation more effective, it is also important to understand individuals’ perception of these goals and gain their support to help transform these goals into specific and actual actions. Furthermore, as the SDGs are a proposed agenda that will expire in 2030, individuals who commit to implement the SDGs may focus more on actions with immediate outcomes and less on actions with benefits that will only become salient after 2030. Therefore, making progress in building a sustainable future requires both individuals’ immediate support for the SDGs and a long-term commitment by individuals to the needs of future generations.

However, the previous literature on SDGs has not investigated what might affect individuals’ perception of the current SDGs and what might motivate individuals to go beyond the SDGs to care for the needs of future generations. As reminders of death can influence attitudes, motivation, and behavior in various aspects of our lives (Greenberg and Kosloff, 2008), in this paper, we thus explored whether reminding individuals of their own death (i.e., mortality salience) will influence their perceived importance of the SDGs and intergenerational altruism when facing the choice between fulfilling the needs of the current generation and those of future generations.

According to terror management theory (TMT, Pyszczynski et al., 1986; Greenberg et al., 2014), humans are caught in a paradox. On the one hand, humans are similar to other species with a strong biological tendency for self-preservation and continued existence; on the other hand, humans are different from other animals in that they have sophisticated cognitive abilities that make them aware that life is temporary and transient, and they will inevitably die 1 day. This co-existence of the desire for immortality and awareness of their mortality can induce existential anxiety. This anxiety can be alleviated by creating a sense of literal (e.g., believing in an afterlife, heaven) or symbolic (e.g., creating publications, artwork) immortality to either deny or transcend ultimate death. Therefore, when reminded of one’s own death, individuals need ways to help them feel a sense of immortality and defend against existential anxiety.

One way individuals can buffer existential anxiety is through culture. It enables individuals to believe that they are significant and enduring beings in a world of meaning rather than merely animals only doomed to death. Culture functions as an anxiety buffer consisting of two components: one is the worldview, which provides a meaningful and stable conception of reality; the other is self-esteem, which is the belief that one is living up to the norms, values, and standards advocated by their culture (Greenberg et al., 2014). According to TMT, when mortality is made salient, individuals are motivated to adhere to their cultural worldviews and believe they are valuable and meaningful within their own culture (Greenberg et al., 1997). In line with this reasoning, empirical evidence has shown that when reminded of their mortality, individuals are more likely to validate their specific cultural worldviews (Rosenblatt et al., 1989) and show greater favoritism toward their in-group members (Castano et al., 2002). Meanwhile, priming death awareness increases people’s prejudice toward others who hold different cultural worldviews (Greenberg et al., 1990), and motivates aggression toward out-group members (McGregor et al., 1998).

Another way individuals can defend against existential anxiety is by denying that humans are a part of nature. It releases humans from the realm of a mortal nature and offers the hope that human existence is not simply temporary, and death can be transcended (Goldenberg et al., 2000). Thus, according to TMT, when primed with mortality salience, individuals become motivated to distinguish themselves from the rest of nature and show negative attitudes toward nature or animals to facilitate their denial of mortality. This logic is supported by research. For example, Koole and Van den Berg (2005) showed that death reminders reduced the perceived beauty of wilderness. Goldenberg et al. (2001) suggested that as individuals become conscious of their own mortality, they are more likely to support the belief that humans are distinct from animals; moreover, they are more likely to disconnect themselves from other animals and treat animals as resources, commodities, and valued goods that benefit human beings (Marino and Mountain, 2015).

Mortality salience not only motivates individuals to behave in ways that are consistent with social norms in their own culture, but also induces individuals to follow the values or norms that are made salient by specific contexts (Jonas et al., 2008). Thus, the effects of mortality salience on attitudes and behavior depends on whether a specific value, norm, identity, or worldview is salient. Taking environmental protection for example, mortality salience causes individuals to be less concerned about the environmental impact of land development unless they derive self-worth from their environmental actions (Vess and Arndt, 2008). Pro-environmental behavior that reduces waste of resources, such as using reusable instead of disposal cups, is induced by mortality salience only when a pro-environmental norm is salient (Fritsche et al., 2010). In addition, perceptions of existential threats result in decreasing biocentric motivation and biospheric concern when environmental identity is low (Fritsche and Häfner, 2012). And for individuals who hold a conservative environmental worldview, mortality salience does not enhance any collective eco-guilt even when these individuals realize the failure of society to meet pro-environmental standards. In comparison, when those individuals who strongly endorse environmental values have those values made salient, mortality salience increases eco-guilt (Harrison and Mallett, 2013).

In addition, intergenerational altruism could serve as another way to repress death-related anxiety. The reason behind this rationale is that intergenerational altruism extends individuals’ existence into the future and helps them to achieve the goal of symbolic immortality by facilitating a psychological connection between the self and others in the future and establishing a legacy to create a lasting impact on others (Wade-Benzoni and Tost, 2009). This rationale was supported by the research of Wade-Benzoni et al. (2012), who found that when making intergenerational decisions about donating money and allocating resources, priming death awareness can trigger individuals’ legacy motivations and lead them to donate more money and allocate more resources to others in the future.

While the prior research has examined the impacts of mortality salience on individuals’ reactions to in-group and out-group members, human-nature relationships, environmental protection, and intergenerational decisions in terms of donating money and allocating resources to future others, we do not yet know whether mortality salience affects individuals’ perceived importance of the SDGs and intergenerational decisions in terms of caring for the needs of future generations. The purpose of this paper, therefore, was to explore the effects of mortality salience on individuals’ perceived importance of the SDGs and intergenerational altruism.

## Materials and Methods

A survey embedded randomized control experiment was conducted to test the effects of mortality salience on intergenerational altruism and the perceived importance of the SDGs. The experiment was conducted in August of 2016.

Participants were recruited from the online survey platform Amazon Mechanical Turk (MTurk). Samples collected via MTurk have been widely used in numerous survey and experiment studies (Paolacci et al., 2010). All participants indicated informed consent electronically, were guaranteed anonymity and allowed to discontinue the study at any time. The study was reviewed and approved by the Ethics Review Committee of Nankai University.

### Participants

A total of 398 MTurk workers located in the United States with a historical approval HIT rate over 90% consented to participate in this study for pay. However, 95 of these participants dropped out: 23.6% (30/127) in the mortality salience condition, 25.2% (34/135) in the dentist visit condition, and 22.8% (31/136) in the neutral condition. A chi-square test showed that the attrition rates across the three conditions were not significantly different, χ^2^(2) = 0.22, *p* = 0.90. All of the 95 quitters left either without answering any questions or with the writing task blank but several subsequent questions answered. The rest of the 303 participants completed the survey. Further, 4 of the 303 participants did not follow the instruction for the writing tasks, and they only answered using simple words like “no ideas,” “no,” and “yes.” The results were consistent when these cases were included/excluded in the analysis. In this paper, we report on results where we excluded these cases.

Of these 299 participants (*M*_age_ = 35.02, *SD* = 11.74), 46% were men, 83% were Caucasian, 45% had children, and 20% attended religious service every week. In term of education attainment and occupation, 73% had college or a higher education degree and 15% worked in hospitals. With regard to socioeconomic status, 54% thought they lived at the average level of socioeconomic status and 13% felt they lived above the average.

To determine if our design was adequately powered, we selected “*Post hoc*: Compute achieved power” for the “Type of power analysis” under “ANOVA: Fixed effects, omnibus, one-way” in G^∗^power software. By entering *f* = 0.37, alpha = 0.05, total sample size = 299, and number of groups = 3, the calculated power that this design has to detect a significant effect that is of the same magnitude as the estimate from the meta-analysis by Burke et al. (2010) is.99.^1^ Thus, the design was adequately powered.

### Design and Procedures

This study used a one-factor between-subject experimental design. Participants were told that the study consisted of several short and independent parts. Part A, which was actually the experimental manipulation section, introduced a mental simulation task. Participants were randomly assigned to one of the following three experimental conditions: mortality salience condition, neutral condition, and dentist visit condition. In the mortality salience condition, participants (*n* = 95) were asked to think of their own death in the future by responding to two questions with no time limit: (1) “Briefly describe the emotions that the thought of your own death arouses in you” and (2) “Jot down, as specifically as you can, what you think will happen to you as you physically die and once you are physically dead.” This manipulation is adopted from Greenberg et al. (1997). In the neutral condition, participants (*n* = 105) were asked to describe a typical grocery shopping experience in their lives. Writing about grocery shopping has been used as a control condition in other areas of research (e.g., attachment theory; Carnelley and Rowe, 2007).

Following the suggestion of the previous literature on mortality salience, we included a comparison condition of dentist visit (Fritsche et al., 2010) to rule out an alternative explanation that the effect of mortality salience on our dependent measures was caused by the negative mood associated with death thoughts. Specifically, we asked participants in the dentist visit condition (*n* = 99) to think about the experience of seeing a dentist by responding to two questions with no time limit: (1) “Briefly describe the emotions that the thought of going to the dentist arouses in you” and (2) “Jot down, as specifically as you can, what do you think will happen to you physically at the dentist.” As previous research has found that levels of negative affect are similar when one thinks about visiting the dentist vs. when one thinks about death (Harrison and Mallett, 2013), including this dentist visit condition can help us further identify the unique effect of mortality salience.

Immediately after that the mental simulation task, all participants reported their momentary mood on the most widely used for measuring affective experience: the Positive and Negative Affect Schedule (PANAS) scale (Watson et al., 1988). It is a 5-point scale (1 = not at all, 5 = extremely) and consists of 10 items that measure positive mood (interested, excited, strong, enthusiastic, proud, alert, inspired, determined, attentive, active; α = 0.91) and 10 items that measure negative mood (distressed, upset, guilty, scared, hostile, irritable, ashamed, nervous, jittery, afraid; α = 0.93).

Then, all participants entered Part B, a word search puzzle used as a filler task for the mortality salience manipulation. The participants were asked to find and write down at least three words that were hidden in an 8 × 7 matrix. The task, which required about 1 min to finish, was added to create a longer delay (Pyszczynski et al., 1999), because prior research suggests that a longer time delay between the mortality salience manipulation and the dependent measures is associated with a larger effect of mortality salience (Burke et al., 2010).

Finally, ostensibly as a third unrelated Part C, all participants responded to the questions that measured their attitude toward intergenerational altruism and the perceived importance of the SDGs. The detailed measures are presented in the next subsection. Demographic information was collected at the end of the survey, including age, gender, education attainment, race, whether they attended religious service or not, whether they had children or not (which might affect participants’ reaction to intergenerational altruism), whether they worked in a hospital or not (which might affect participants’ reaction to thinking of death), and subjective socioeconomic status.

### Dependent Measures

Our dependent measures included two parts. The first part was to measure intergenerational altruism. Specifically, all participants read a text that said “sustainable development has been defined as development that meets the needs of the present without compromising the ability of future generations to meet their own needs. It often embraces a triple bottom line combining the interest of the present generation and the interest of the future generations. However, sometimes there are competing priorities. In your opinion, whose needs should be given higher priority?” Participants were asked to make a choice between two options: A = the needs of our current generation, B = the needs for future generations.

The second part measured the perceived importance of the 17 SDGs. All participants first read a brief introduction to the sustainable development agenda and the 17 SDGs as follows: “The United Nations announced an agenda for sustainable development last year, following an inclusive process of intergovernmental negotiations. As a plan of action, this agenda will be implemented by all countries and stakeholders. The agenda consists of 17 sustainable development goals, which are listed below.” Then, participants were asked to indicate how important they think each of these goals was on 5-point Likert scales (1 = not at all important, 5 = extremely important).

The order of the two dependent measurements was counterbalanced. As order of presentation had no effect on our results, we did not include this variable in the subsequent analyses.

## Results

### Effect of Mortality Salience on Mood

To examine whether mortality salience affects participants’ positive and negative moods and rule out mood as an alternative explanation for our effects, we separately conducted two one-way ANOVAs for positive mood (averaged the score of 10 positive mood items) and negative mood (averaged the score of 10 negative mood items). The results showed that there was no significant difference for the three experimental conditions in terms of positive mood, *F*(2,296) = 0.36, *p* = 0.70, η_p_^2^ = 0.002, but there was a significant difference for negative mood, *F*(2,296) = 9.26, *p* < 0.01, η_p_^2^ = 0.06.

Pairwise comparisons using Bonferroni method suggested that when compared to the neutral condition (*M*_neutral_ = 1.39, *SD* = 0.73), a significant difference in the participants’ negative mood was produced by both mortality salience condition (*M*_mortality_ = 1.72, *SD* = 0.77), *t*(297) = 3.55, *p* < 0.01, and dentist visit condition (*M*_dentist_ = 1.78, *SD* = 0.75), *t*(297) = 3.90, *p* < 0.01. However, there was no significant difference in the participants’ negative mood between the mortality salience and dentist visit conditions, *t*(297) = -0.1, *p* > 0.99.

These results suggest that there was increased negative mood in the mortality salience condition compared to the neutral condition. However, the negative mood in the mortality salience and the dentist visit conditions were not significantly different. This similar level of negative mood in the mortality salience and the dentist visit conditions allows us to examine the specific effects of mortality salience by comparing these two conditions.

### Effect of Mortality Salience on Intergenerational Altruism

A chi-square test was conducted to examine the effect of experimental conditions on intergenerational altruism. The result showed that the experimental conditions had a significant effect on the participants’ judgment regarding the priority between the needs of the current generation and the needs of future generations, χ^2^(2) = 6.26, *p* = 0.04, Cramer’s *V* = 0.15.

Pairwise comparisons using partition of chi-square method (running separate chi-square tests comparing each condition to one another) indicated that making mortality salient caused a higher percentage of participants (63.2%) to place a higher priority on the needs of future generations when compared to the neutral condition (45.7%), χ^2^(1) = 6.11, *p* = 0.013^2^; however, it did not induce a significantly higher percentage of participants to place a higher priority on the needs of future generations when compared to the dentist visit condition (51.5%), χ^2^(1) = 2.68, *p* = 0.101. In addition, priming the dentist visit, when compared to the neutral condition, did not significantly increase the percentage of participants who chose the needs of future generations as a greater priority, χ^2^(1) = 0.69, *p* = 0.41. Detailed information on these result are shown in **Table 1** below.

**Table 1:** Comparing intergenerational altruism across the three conditions.

Intergeneration altruism	Mortality salience *n* = 95		Dentist visit *n* = 99		Neutral *n* = 105	
		
	Count	%	Count	%	Count	%
Needs of current generation	35	36.8	48	48.5	57	54.3
Needs of future generations	60	63.2_a_	51	51.5_a_	48	45.7_b_


*Percentages in the rows without a common subscript differed at p < 0.05 between conditions.*

Consistent results were produced by using binary logistic regression to investigate the effect of mortality salience on intergenerational altruism (These results are available in the **Supplementary Material**).

### Factor Analysis and the Effect of Mortality Salience on the Perceived Importance of the SDGs

While the 17 SDGs were combined as an idea serving the political agenda of building a sustainable future, it is unclear whether these goals also converged as one factor or not in the minds of the participants. Thus, we performed an exploratory factor analysis to further explore the underlying relationship between these 17 SDGs. Following the recommendations of Costello and Osborne (2005), we first selected principle axis factoring as the factor extraction method since some of the SDG items, such as ending poverty (SDG1), gender equality (SDG5), and combating climate change (SDG13), had negatively skewed distributions. Then, we chose direct oblimin rotation instead of varimax rotation method as we expected the underlying factors to be correlated.

The result yielded two factors with the eigenvalues > 1 and a high correlation coefficient of these two factors (*r* = 0.77, *p* < 0.01), justifying the rotation approach we chose. The first unrotated factor accounted for 49.08% of the total variance, and the second unrotated factor accounted for an additional 3.70%. Of the 17 SDGs, 12 items loaded on the first factor (all loadings ≥ 0.49) with weak cross-loadings on the second factor (all loadings ≤ 0.28) while 5 items loaded on the second factor (all loadings ≥ 0.45) with weak cross-loadings on the first factor (all loadings ≤ 0.29). As the 12 items in the first factor predominately related to social issues and the 5 items in the second factor were associated with ecological issues, we labeled them as the socially related SDGs (α = 0.95) and the ecologically related SDGs (α = 0.86), respectively. (See **Supplementary Table S3** in the **Supplementary Material** for the detailed information about items in each factor and their loadings).

To examine the effect of mortality salience on the perceived importance of the socially related SDGs and the ecologically related SDGs, we created two indexes by averaging the scores of their respective items. Then, two one-way ANOVAs with each factor as a dependent variable were performed separately. The results showed that the experimental conditions had a significant effect on the perceived importance of the socially related SDGs, *F*(2,296) = 4.46, *p* = 0.01, η_p_^2^ = 0.03, and a marginally significant effect on the perceived importance of the ecologically related SDGs, *F*(2,296) = 2.98, *p* = 0.054, η_p_^2^ = 0.02.

Pairwise comparisons using Bonferroni method for the perceived importance of the socially related SDGs indicated that participants in the mortality salience condition (*M*_mortality_= 3.73, *SD* = 0.85) had a significantly lower mean than those in the dentist visit condition (*M*_dentist_ = 3.99, *SD* = 0.66), *t*(297) = 2.56, *p* = 0.03, and those in the neutral condition (*M*_neutral_ = 4.00, *SD* = 0.57), *t*(297) = 2.60, *p* = 0.03. However, participants in the neutral condition and those in the dentist visit condition did not significantly differ in the perceived importance of the socially related SDGs, *t*(297) = -0.1, *p* > 0.99.

Similar pairwise comparisons for the perceived importance of the ecologically related SDGs showed that participants in the mortality salience condition (*M*_mortality_ = 3.81, *SD* = 0.88) did not significantly differ from those in the dentist visit condition (*M*_dentist_ = 4.01, *SD* = 0.71), *t*(297) = 1.82, *p* = 0.21, but they had a marginally significant lower mean than those in the neutral condition (*M*_neutral_ = 4.07, *SD* = 0.70), *t*(297) = 2.30, *p* = 0.053. Further, participants in the neutral condition and those in the dentist visit condition did not significantly differ in the perceived importance of the ecologically related SDGs, *t*(297) = -0.45, *p* > 0.99.

**FIGURE 1:**
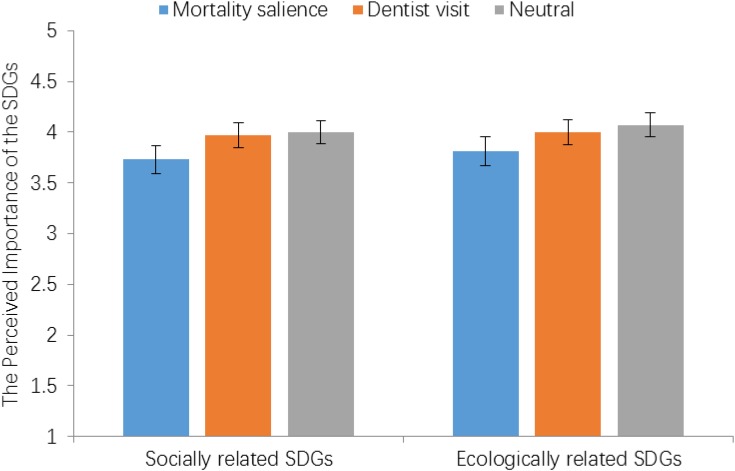
The effects of experimental conditions on the perceived importance of two factors of the SDGs. Error bars represent 95% confidence intervals.

To facilitate the interpretation of the results, **Figure 1** illustrates the effects of experimental conditions on the perceived importance of the socially related SDGs as well as the ecologically related SDGs.

### Additional Analyses

Additional analyses were performed to explore whether any of our measured demographical variables influenced the observed effects.

The effect of gender was first examined^3^. A 2 (gender) × 3 (experimental conditions) ANOVA on the perceived importance of the socially related SDGs indicated a significant main effect of experimental conditions, *F*(2,293) = 4.72, *p* = 0.01, η_p_^2^ = 0.08, a significant main effect of gender, *F*(1,293) = 23.95, *p* < 0.01, η_p_^2^ = 0.08, and a marginally significant interactive effect of gender and experimental conditions, *F*(2,293) = 2.82, *p* = 0.06, η_p_^2^ = 0.02. Simple effects analyses showed that the perceived importance of the socially related SDGs among the three conditions were significantly different for men, *F*(2,293) = 7.20, *p* < 0.01, η_p_^2^ = 0.08, but they were not significantly different for women, *F*(2,293) = 0.13, *p* = 0.88, η_p_^2^ = 0.001. Further pairwise comparisons with Bonferroni-adjusted method revealed that men under the mortality salience condition perceived the socially related SDGs (*M*_mortality_ = 3.40, *SD* = 0.90) as significantly less important than men under the dentist visit condition (*M*_dentist_ = 3.85, *SD* = 0.75), *t*(297) = 3.21, *p* < 0.01, and the neutral condition (*M*_netural_ = 3.86, *SD* = 0.61), *t*(297) = 3.29, *p* < 0.01. However, men under the dentist visit and the neutral conditions did not differ significantly in the perceived importance of the socially related SDGs, *t*(297) = 0.04, *p* = 0.97.

A similar ANOVA on the perceived importance of the ecologically related SDGs showed a significant main effect of experimental conditions, *F*(2,293) = 3.10, *p* = 0.046, η_p_^2^ = 0.04, a significant main effect of gender, *F*(1,293) = 10.76, *p* < 0.01, η_p_^2^ = 0.04, and a marginally significant interactive effect of gender and experimental conditions, *F*(2,293) = 2.48, *p* = 0.09, η_p_^2^ = 0.02. Simple effects analyses indicated that the perceived importance of the ecologically related SDGs among the three conditions significantly differed for men, *F*(2,293) = 4.70, *p* = 0.01, η_p_^2^ = 0.03, but they did not significantly differ for women, *F*(2,293) = 0.61, *p* = 0.54, η_p_^2^ = 0.004. Further pairwise comparisons with Boniferroni-adjusted method suggested that mortality salience induced men to perceive the ecologically related SDGs (*M*_mortality_ = 3.58, *SD* = 0.91) as significantly less important compared to the neutral condition (*M*_neutral_ = 4.06, *SD* = 0.76), *t*(297) = 3.13, *p* < 0.01, but there was no significant difference when compared to the dentist visit condition (*M*_dentist_ = 3.80, *SD* = 0.75), *t*(297) = 1.31, *p* = 0.18. In addition, men in the dentist visit condition perceived the ecologically related SDGs as marginally significantly less important than men in the neutral condition, *t*(297) = 1.67, *p* = 0.095.

The effects of other variables, including race, whether the participant had children or not, whether the participant worked in hospitals or not, religious service attendance, education attainment, and subjective socioeconomic status were also investigated. These results suggested that none of these factors had main and interactive effects on the perceived importance of the socially related SDGs and the ecologically related SDGs.

## Discussion

This paper explored the effects of mortality salience on intergenerational altruism and individuals’ perceived importance of the SDGs. We found that mortality salience induced participants to care more about the needs of future generations only when compared to the neutral condition. We also found mortality salience reduced participants’ perceived importance of the socially related SDGs when compared to both the dentist visit and the neutral conditions, and mortality salience decreased participants’ perceived importance of the ecologically related SDGs only when compared to the neutral condition.

### Effect of Mortality Salience on Intergenerational Altruism

Our research revealed that when compared to the neutral condition (writing about a typical grocery shopping experience), mortality salience can promote intergenerational altruism (i.e., placing a higher priority on the needs of future generations). Our research also indicated that participants in the mortality salience condition did not significantly differ in their prioritization of the needs of future generations compared to those in the dentist visit condition. Although the current study does not provide conclusive evidence for the unique effect of mortality salience on intergenerational altruism, the fact that the percentage of participants who chose the needs of future generations was the highest in the mortality salience condition is suggestive. The reason behind this potential positive effect of mortality salience might be that acting on behalf of future generations helps individuals achieve the goal of outlasting their own existence, thereby repressing death-related anxiety (Wade-Benzoni and Tost, 2009).

Our finding was consistent with the research of Wade-Benzoni et al. (2012), which showed that mortality salience motivates individuals to behave generously to benefit future others when compared to the control condition (writing about the author’s writing style after reading a newspaper article). However, there are two differences between our research and their paper in terms of research design. First, their paper asked participants to make intergenerational decisions such as donating money and allocating resources between present and future others, but we directly asked participants to choose whether the current or the future generation’s needs should be a higher priority. Second, their paper included only two conditions (mortality salience vs. control condition). In our design, we added a dentist visit condition. Our research, by using an alternative measurement and a three-condition design, provided partial support for the argument that intergenerational altruism might serve as a way for individuals to buffer anxiety when they are reminded of their own mortality (Wade-Benzoni and Tost, 2009), and somewhat extended the effect of mortality salience on self-protective altruism (Hirschberger et al., 2008) to intergenerational altruism.

### Effect of Mortality Salience on the Perceived Importance of the SDGs

As for the effect of mortality salience on the perceived importance of the SDGs, our results showed that mortality salience reduced the perceived importance of the socially related SDGs when compared to both the dentist visit and neutral conditions. A potential explanation for this negative effect of mortality salience might be that individuals want to support their own worldview and not follow the global agenda, because the global agenda might blur the cultural differences that make each culture unique. When perceiving the importance of those SDGs that seek to solve global social issues and promote global benefits, the participants, who were American in our study, might have seen these goals as infringing on their own cultural worldview and freedoms. Consequently, the perceived importance of the socially related SDGs was reduced when participants were reminded of their own mortality.

This finding partially echoed the research of Jonas et al. (2002), which showed that mortality salience increased participants’ tendency to help poor people, but only for those in the participants’ own country. In other words, mortality salience had no effect on helping out-groups when in-groups and out-groups were distinguished; however, as shown in our research, mortality salience had a negative effect on the perceived importance of social issues that included helping others when in-groups and out-groups were combined. This result may contribute to the literature that suggests reminders of mortality increase negative evaluations toward out-group members or dissimilar others (Greenberg and Kosloff, 2008).

Our results also indicated that mortality salience led to marginally significant reductions in the perceived importance of the ecologically related SDGs when compared to the neutral condition. However, it should be noted that participants in the mortality salience and the dentist visit condition did not significantly differ on the perceived importance of the ecologically related SDGs. Although these results do not provide conclusive evidence for the unique effect of mortality salience on the perceived importance of the ecologically related SDGs, the fact that the perceived importance of the ecologically related SDGs was the lowest in the mortality salience condition is suggestive. The potential negative effect of mortality salience on the perceived importance of the ecologically related SDGs can be explained by the reasoning that separating humans from nature could make individuals uphold their belief that they are not subject to the natural laws of death and decay (e.g., Goldenberg et al., 2000). The ecologically related SDGs included cues of wild animals and nature, which may remind individuals of their own biological identity and mortal nature. As a result, when mortality was made salient and participants were asked to perceive the importance of the SDGs that aim to protect endangered animals and the natural environment, they would deny their creatureliness and perceive the ecologically related SDGs as less important.

In addition, our results revealed that gender and experimental conditions produced marginally significant interactive effects on the perceived importance of the socially related SDGs and the ecologically related SDGs. Specifically, mortality salience led men (but not women) to perceive the socially related SDGs as significantly less important than men both in the dentist visit and the neutral conditions. This result suggested that gender moderates the effect of mortality salience on the perceived importance of socially related SDGs. Mortality salience also caused men (but not women) to perceive the ecologically related SDGs as significantly less important than men in the neutral condition, but not in the dentist visit condition. Thus, this result only provided partial support for a potential moderating role of gender on the effect of mortality salience on the perceived importance of ecologically related SDGs. The (potential) moderating role of gender may be explained as follows: men tend to hold less universalistic values (Schwartz and Rubel, 2005) and pro-environmental attitudes (Gifford and Nilsson, 2014). When men are reminded of their mortality, they may become more entrenched in their worldview, and their perceptions of the importance of the socially related SDGs and the ecologically related SDGs decrease.

### Limitations and Future Directions

Despite the efforts made in this research, there are limitations that can potentially be addressed through further research. As mentioned above, the effects of mortality salience on attitudes, motivation, and behavior involving in-group vs. out-group members may differ (Jonas et al., 2002), and the effects of morality salience may depend on the activation of specific norms and values (Jonas et al., 2008). Future research could examine the moderating role of individual’s group identity (e.g., Chinese vs. American) and self-transcendence values (Schwartz, 1994) on the effect of mortality salience on the perceived importance of the SDGs. In addition, as implementing SDGs may require large government investment and incur debt in the present time, individuals who are more conservative in their political ideology may view achieving some of these SDGs as potentially burdening future generations. Thus, the political orientation of individuals should be investigated as a potential moderator in future research. Moreover, our results might have been influenced by individuals’ perceptions of the UN. Specifically, individuals who do not like the UN may not support plans proposed by the UN and may have given less importance to these SDGs. So, it is also important to explore whether changing the fact that the UN is the source of these goals will make our results different.

In terms of manipulations, our priming of mortality salience only asked the participants to write down their thoughts of death. Future research could improve on this method by implementing field experiments in special event situations that will potentially remind people of their mortality (e.g., natural disasters, Västfjäll et al., 2014). Furthermore, writing about the experience of grocery shopping touched on issues like food security, accessibility, and availability and thinking about visiting the dentist involved issues related to health. These two writing tasks might influence individuals’ perceived importance of those SDGs that focus on food security and healthy lives, so future research might utilize different comparison conditions to try to replicate our results. As for the measurement of intergenerational altruism, it was a binary choice. While this choice is straightforward, future research could operationalize this concept through simulated decision scenarios (e.g., the dictator game).

With regard to sample selection, participants in this study were recruited from MTurk and lived in the United States. Future research should recruit participants from different countries to test whether and how individuals with various cultural worldviews perceive the importance of the SDGs in similar or different ways. In addition, we did not identify whether the participants in our study had been exposed to similar experimental manipulations before. Further research could check whether the MTurk workers who participated are naïve to mortality salience research and whether previous experience might affect their responses on the outcomes of interest (Chandler et al., 2014).

## Author Contributions

SH and XZ designed the study and oversaw all aspects of the study implementation and data collection. NZ wrote the first manuscript. JZ reviewed the first version of the manuscript and made substantial contributions to the interpretation of the data. XZ carried out the statistical analyses. NZ and SH conceptualized and oversaw analyses, and made substantial contributions to the interpretation of the data. All authors made critical revisions to the final draft and agree to be accountable for all aspects of the work in ensuring that questions related to the accuracy or integrity of any part of the work are appropriately investigated and resolved.

## Conflict of Interest Statement

The authors declare that the research was conducted in the absence of any commercial or financial relationships that could be construed as a potential conflict of interest.

## Footnotes

1 Originally, the “ANOVA: Fixed effects, special, main effects and interaction” power analysis in G^∗^Power was used to calculate sample size. Considering a meta-analysis of terror management research that reported a medium effect size (*r* = 0.35) for mortality salience effect (Burke et al., 2010), we set up the effect size *f* = 0.35, and we set up α = 0.05, power = 0.95, df = 16, and the number of groups = 3. Then, we obtained a sample size of 245. Due to concerns about the difference in quality between data collected online and data collected in the laboratory, we elected to collect a larger sample of participants than the one we had calculated. However, during the review process, the reviewer pointed out that we did not convert the effect size r to the effect size *f* and chose a wrong value of df. We thanked the reviewer and acknowledged the errors. As the analytic approach was changed due to reviewer feedback, the power for the obtained sample size was calculated with the errors fixed.

2 This *p*-value was still below the corrected alpha level (0.05/3) when we controlled for type I error.

3 This was a *post hoc* analysis. We thanked the reviewer proposed that gender might be a moderator given that some items of the SDGs are about women’s rights.
