# Supplementary material for: The Impact of Mortality Salience on Intergenerational Altruism and the Perceived Importance of Sustainable Development Goals
Source: Front Psychol. 2018 Aug 3;9:1399. doi: 10.3389/fpsyg.2018.01399 (PMC6085722; doi:10.3389/fpsyg.2018.01399)
Supplement: Supplementary file 1 [file Table_1.DOC]

**The Impact of Mortality Salience on Intergenerational Altruism and the Perceived Importance of Sustainable Development Goals**

Saiquan Hu, Xiaoying Zheng, Nan Zhang, Junming Zhu

**Effect of mortality salience on intergenerational altruism**

A binary logistic regression was conducted to examine the effect of experimental conditions on participants’ intergenerational altruism. The dependent variable was a dummy variable and coded as 0 = the needs of our current generation, 1=the needs of the future generations. We used two dummy variables (*D1, D2*) to represent mortality salience condition (*D1 = 1, D2 = 0*), dentist visit condition (*D1 = 0, D2 = 1*), and neutral condition (*D1 = 0, D2 = 0*).

The results showed that the effect of mortality salience was significant (*β* = .711, *p* = .014), whereas the effect of dentist visit was not significant (*β* = .314, *p* = .238). Stated another way, priming mortality salience, as compared to the neutral condition, caused a larger percentage of participants to place a higher priority on the needs of future generations (mortality salience condition: 63.2% vs. neutral condition: 45.7%). However, priming dentist experience, as compared to the neutral condition, did not significantly increase the percentage of participants that chose the needs of future generations as a greater priority (dentist visit condition: 51.5% vs. neutral condition: 45.7%).

These results were also consistent when demographical variables were controlled, including age, gender (male = 1, female = 0), race (Caucasian = 1, non-Caucasian = 0), religious service attendance (yes = 1, no = 0), subjective socioeconomic status (below average = 1, average =2, and above average =3), education attainment (below college = 1, college = 2, and above college = 3), whether the participant had children or not (yes = 1, no = 0), and whether the participant worked in hospitals or not (yes =1, no = 0). Detailed results can be found in Table S1.

Table S1. Effect of mortality salience and dentist visit conditions

on intergenerational altruism

|  | Model 1 | Model 2 |
| --- | --- | --- |
| **Independent variable** |  |  |
| Mortality salience | .711* | .751* |
| Dentist visit | .314 | .341 |
| **Control variables** |  |  |
| Age |  | -.018 |
| Gender |  | .030 |
| Race |  | -.169 |
| Religious service attendance |  | -.351 |
| Subjective socioeconomic status |  | -.003 |
| Education attainment |  | -.039 |
| Whether the participant had children or not |  | .078 |
| Whether the participant worked in hospitals or not |  | -.434 |
| Cox & Snell *R^2^* | .020 | .041 |
| Nagelkerke *R^2^* | .027 | .055 |

*Notes:* N = 299；*indicates *p* < .05.

We also performed an additional binary logistic regression to compare mortality salience condition to the dentist visit condition. We treated mortality salience as the reference group in this analysis. The results showed that the effect of dentist visit condition on the intergenerational altruism was not significant (*β* = -.397, *p* = .175). Stated another way, priming dentist visit, as compared to the mortality salience condition, did not significantly increase the percentage of participants that chose the needs of future generations as a greater priority (dentist visit condition: 51.5% vs. mortality salience condition: 63.2%). These results were consistent when control variables were all included. Detailed results can be found in Table S2.

Table S2. Effect of the neutral and dentist visit conditions

on intergenerational altruism

|  | Model 1 | Model 2 |
| --- | --- | --- |
| **Independent variable** |  |  |
| Neutral condition | -.711* | -.751* |
| Dentist visit | -.397 | -.410 |
| **Control variables** |  |  |
| Age |  | -.018 |
| Gender |  | .030 |
| Race |  | -.169 |
| Religious service attendance |  | -.351 |
| Subjective socioeconomic status |  | -.003 |
| Education attainment |  | -.039 |
| Whether the participant had children or not |  | .078 |
| Whether the participant worked in hospitals or not |  | -.434 |
| Cox & Snell *R^2^* | .020 | .041 |
| Nagelkerke *R^2^* | .027 | .055 |

*Notes:* N = 299；*indicates *p* < .05.

**Table S3. Exploratory Factor analysis of the 17 items of** SDGs

| Factors | The 17 items of SDGs | Loading on Factor 1 | Loading on factor 2 |
| --- | --- | --- | --- |
| The socially related SDGs | 1. End poverty in all its forms everywhere. | **0.68** | 0.14 |
|  | 2. End hunger, achieve food security and improved nutrition, and promote sustainable agriculture. | **0.55** | 0.23 |
|  | 3. Ensure healthy lives and promote well-being for all at all ages. | **0.77** | -0.01 |
|  | 4. Ensure inclusive and equitable quality education and promote lifelong learning opportunities for all. | **0.83** | -0.07 |
|  | 5. Achieve gender equality and empower all women and girls. | **0.59** | 0.07 |
|  | 7. Ensure access to affordable, reliable, sustainable, and modern energy for all. | **0.49** | 0.28 |
|  | 8. Promote sustained, inclusive, and sustainable economic growth, full employment, and decent work for all. | **0.85** | -0.12 |
|  | 9. Build resilient infrastructure, promote inclusive and sustainable industrialization, and foster innovation. | **0.55** | 0.1 |
|  | 10. Reduce inequality within and between countries. | **0.73** | -0.07 |
|  | 11. Make cities and human settlements safe, resilient and sustainable. | **0.65** | 0.14 |
|  | 16. Promote peaceful and inclusive societies for sustainable development, and build inclusive institutions at all levels. | **0.77** | -0.02 |
|  | 17. Strengthen the means of implementation and revitalize the global partnership for sustainable development. | **0.57** | 0.04 |
| The ecologically related SDGs | 6. Ensure availability and sustainable management of water and sanitation for all. | 0.29 | **0.45** |
|  | 12. Ensure sustainable consumption and production patterns. | 0.19 | **0.52** |
|  | 13. Take urgent action to combat climate change and its impacts. | 0.13 | **0.59** |
|  | 14. Conserve and sustainably use the oceans, seas, and marine resources for sustainable development. | -0.08 | **0.83** |
|  | 15. Protect, restore, and promote sustainable use of terrestrial ecosystems, halt land degradation and biodiversity loss. | 0.02 | **0.82** |
